# Combination of fluorescent reagents with 2-(4-aminophenyl) benzothiazole and safranin O was useful for analysis of spore structure, indicating the diversity of *Bacillales* species spores

**DOI:** 10.3389/fmicb.2025.1603957

**Published:** 2025-06-25

**Authors:** Ritsuko Kuwana, Kiyoshi Ito, Hiromu Takamatsu

**Affiliations:** Faculty of Pharmaceutical Sciences, Setsunan University, Hirakata, Japan

**Keywords:** spore structure, spore coat, fluorescent staining, safranin O, spore diversity, *Bacillales*

## Abstract

**Background:**

Safranin O is commonly used for the gram staining of bacteria and fluorescent staining of plant tissues. We aimed to perform a more detailed structural analysis of bacterial spores by analyzing the staining pattern of safranin O, together with a combination of other fluorescence probes, including 2-(4-aminophenyl) benzothiazole (APBT).

**Methods:**

We stained spores from six *Bacillales* species, including *Bacillus subtilis, B. licheniformis, Niallia circulans, Brevibacillus brevis, Lysinibacillus sphaericus*, and *Paenibacillus polymyxa*, with safranin O and APBT and observed them using fluorescence microscopy. We also performed comparative analysis using other fluorescent reagents, including auramine O, rhodamine B, thioflavin T, and congo red. Additionally, the localization of spore proteins was analyzed by green fluorescent protein (GFP)-fused strains and spore-forming-defective mutant strains of *B. subtilis*.

**Results:**

Fluorescence microscopy analysis revealed that safranin O exhibits two distinct fluorescence peaks, green and red, in *Bacillales* species in different regions of the spore structure, indicating the complexity and diversity within the spore structures. APBT fluorescence co-localized with specific spore structures and aligned with the GFP fused strains, which were used as marker proteins for the spore structural components, such as the outermost spore layer (crust), inner spore coat, cortex, and inner spore membrane. Safranin O red fluorescence was detected near the inner spore coat, congo red, and thioflavin T fluorescence. In contrast, the green fluorescence regions were similar to those identified by APBT, auramin O, and rhodamine B. Spore morphogenesis-deficient mutants, including *spoIVA* and *cotE*, exhibited altered fluorescence patterns with APBT and safranin O, indicating abnormal spore structures and staining of forespore periphery.

**Conclusion:**

These findings show that safranin O produces distinct red and green fluorescence patterns in bacterial spores. The combined use of safranin O and other fluorescent probes with fluorescence microscopy and GFP fusion proteins offers a powerful approach for visualizing and analyzing bacterial spore structures. The present study on *Bacillales* spores may have broad applications in environmental microbiology, food safety, and biosecurity. It may provide a framework for rapid detection of spore-forming bacteria during industrial fermentation and antimicrobial drug development.

## 1 Introduction

Members of the phylum *Bacillota*, particularly *Bacillus* and *Clostridium*, produce dormant spores that are highly resistant to various physical and chemical cytotoxic stressors (Setlow, 2014). *Bacillus subtilis* is a well-studied model organism for spore-forming bacteria, and its gene expression profile and morphogenetic mechanisms throughout the sporulation process, from initiation to spore maturation, have been extensively investigated.

During vegetative growth, *B. subtilis* undergoes binary fission to produce identical daughter cells. However, under specific environmental conditions, it switches to asymmetric division to initiate sporulation (Piggot and Coote, 1976; Stragier and Losick, 1996). This process creates two unequal-sized daughter cells, a smaller prespore and a larger mother cell, with distinct gene expression profiles (Veening et al., 2008; Errington, 1993, 2003). The prespore is engulfed by the mother cell to form the forespore, which is enclosed by two membrane layers, an inner and an outer spore membrane, as well as a peptidoglycans-rich cortex that develops between these membranes (Errington, 1993, 2003). Within the inner spore membrane, the cytoplasm of the forespore contains chromosomal DNA termed the core of mature spores (Errington, 1993, 2003). Surrounding the outer spore membrane, a proteinaceous spore coat structure forms, consisting of the inner and outer coats and a surface-associated crust (Errington, 1993, 2003; Driks, 1999; Bartels et al., 2019; Shuster et al., 2019; Henriques and Moran, 2007). The crust is analogous to the exosporium present in the spores of *B. megaterium* and other bacterial spores (Manetsberger et al., 2018). While the overall spore structure is conserved among spore-forming bacteria, spore coat components and sizes vary across species (Galperin et al., 2012, 2022).

The structures of spore-forming cells and mature *B. subtilis* spores have been elucidated by electron microscopy, atomic force microscopy, and quick-freeze replica electron microscopy (Tang et al., 2007; Driks and Eichenberger, 2016; Jalil et al., 2024). Fluorescent protein-based structural analysis has also been performed to confirm the localization of proteins within the coat, cortex, and core of *B. subtilis* spores (Taoka et al., 2024). Guided by morphogenetic proteins, such as SpoIVA, SpoVID, SafA, and CotE, spore coat and crust proteins form layered structures during mid- to late-stage sporulation (Driks, 1999). Additionally, small acid-soluble spore proteins (SASPs) in the core bind to chromosomal DNA to form ring-like structures (Ragkousi et al., 2000).

To study the structure of spore-forming bacteria such as *B. subtilis, B. cereus, Clostridium botulinum, C. perfringens*, and *C. sporogenes*, various fluorescent dyes, in addition to fluorescent proteins, have been used (Hosomi et al., 2015; Yasugi et al., 2016; Kuwana et al., 2022, 2023, 2024). Fluorescent dyes are particularly useful for visualizing cellular structures that cannot be analyzed with fluorescent proteins and observing bacterial species where the genetic introduction of fluorescent proteins is difficult. Our group has been exploring fluorescent dyes for analyzing cellular structures of spore-forming bacteria and has previously reported fluorescence microscopy studies on *B. subtilis* using 2-(4′-methylaminophenyl) benzothiazole (BTA-1) and 2-(4-aminophenyl) benzothiazole (APBT) for blue fluorescence, auramine O for green fluorescence, and congo red and rhodamine B for red fluorescence (Kuwana et al., 2023). These fluorescent dyes facilitate multicolor staining because they consist of components that have distinct excitation and emission wavelengths (Kuwana et al., 2023).

In this study, we investigated safranin O as a fluorescent dye for staining bacterial spores. Safranin O, which is commonly used for gram staining (Bartholomew and Mittwer, 1952), also binds to acidic lignin polymers in plant cell walls, producing red and green fluorescence in lignin-rich and low lignin regions, respectively (Baldacci-Cresp et al., 2020). Although bacteria lack lignin, their spore structures contain densely packed macromolecules such as proteins and peptidoglycans, some of which are acidic in nature. We hypothesized that safranin O might exhibit distinct fluorescence patterns when bound to specific regions within bacterial spores. To investigate this, we stained wild-type and sporulation-deficient *B. subtilis* mutants as well as various *Bacillales* species and analyzed their fluorescence profiles using microscopy. The study findings may offer a useful tool for bacterial spore structure analysis.

## 2 Materials and methods

### 2.1 Bacterial strains, plasmids, media, and general techniques

The *Bacillales* and *Escherichia coli* strains and the plasmids used in this study are listed in Table 1. *B. subtilis* strains were derivatives of strain 168, and those newly constructed in this study were generated via plasmid DNA transformation and confirmed by polymerase chain reaction (PCR) (Kuwana et al., 2003, 2004, 2006; Takamatsu et al., 2009). *E. coli* strain JM109 was used for plasmid propagation. The oligonucleotides used for PCR amplification are listed in Table 2. To amplify the *cgeA* fragment from the *B. subtilis* 168 chromosome, primers CGEAM490 and CGEA398R were used. The resultant PCR product was digested at the *Bam*HI and *Xho*I sites introduced by the primers and ligated into the *Bam*HI/*Xho*I-digested pGFP7CA vector to create the plasmid pCGEA8GA (Imamura et al., 2011; Table 1). This plasmid was introduced into the *amyE* locus of strain 168 via transformation. Single-crossover transformants were identified by chloramphenicol (5 μg/mL) selection, yielding the CGEA8GA construct (Table 1). Recombinants were confirmed via PCR. Green fluorescent protein (GFP)-fused strains were constructed using overlap extension PCR (Nishikawa and Kobayashi, 2021). To construct strain G-0140, a *gfp*-cat cassette was PCR-amplified from plasmid pCBGFPUV4 using UV4-F and cat-T-R primers (Table 2). The cat-T-R primer contained transcription terminators to prevent read-through. Similarly, to construct strains G-0399 and G-0550, a *gfp*-cat cassette was PCR-amplified from plasmid pCBGFPUV4 using UV4-F and cat-R primers (Table 2). The coding and downstream regions of the target gene were amplified using the primer pairs F1/R1 and F2/R2 (Table 2). The 5′ sequences of R1 and F2 were complementary to those of UV4-F and cat-R or cat-T-R, respectively. The three PCR fragments were fused and used as templates for a second round of PCR with primers F1 and R2. The final PCR products were transformed into strain 168, generating *gfp*-*cat* strains through double-crossover recombination. Transformants were selected on Luria–Bertani (LB; BD Japan Co. Tokyo, Japan) agar plates containing chloramphenicol (5 μg/mL).

**Table 1:** Bacterial strains used in this study.

**Species**	**Strains**	**Genotype**	**References**
*B. subtilis*	168	*trpC2*	1A1 (*Bacillus* Genetic Stock Center)
*B. subtilis*	SIGF5E	*trpC2, sigF* (*spoIIAC*)::pMutin3	Takamatsu et al., 2009
*B. subtilis*	SIGE5E	*trpC2, sigE* (*spoIIGB*)::pMutin3	Takamatsu et al., 2009
*B. subtilis*	SIGG5E	*trpC2, sigG* (*spoIIIG*)::pMutin3	Takamatsu et al., 2009
*B. subtilis*	SIGK5E	*trpC2, sigK* (*spoIVCB*)::pMutin3	Takamatsu et al., 2009
*B. subtilis*	COTE5E	trpC2 *cotE*::pMutin3	Kuwana et al., 2004
*B. subtilis*	GERE5E	*trpC2 gerE*::pMutin3	Kuwana et al., 2003
*B. subtilis*	S4A5E	*trpC2 spoIVA*::pMutin3	Kuwana et al., 2006
*B. subtilis*	CGEA8GA	*trpC2 amyE*::P*_*cgeA*_*-*cgeA-gfp*-H6 *cat*	This work
*B. subtilis*	YEEK8G	*trpC2, yeeK-gfp cat*	Takamatsu et al., 2009
*B. subtilis*	YHCN8G	*trpC2, yhcN-gfp cat*	Takamatsu et al., 2009
*B. subtilis*	G-0140	*trpC2, atpC-gfp cat* terminator	This work
*B. subtilis*	G-0399	*trpC2, alonB-gfp cat*	This work
*B. subtilis*	G-0550	*trpC2, QoxB-gfp cat*	This work
*B. licheniformis*	ATCC14580	*wild type*	American Type Culture Collection (ATCC, Manassas, VA, USA)
*Niallia circulans*	NBRC 13629	*wild type*	NITE Biological Resource Center (NBRC, Tokyo, Japan)
*Brevibacillus brevis*	NBRC 100599	*wild type*	NITE Biological Resource Center (NBRC, Tokyo, Japan)
*Lysinibacillus sphaericus*	NBRC 3526	*wild type*	NITE Biological Resource Center (NBRC, Tokyo, Japan)
*Paenibacillus polymyxa*	NBRC15309	*wild type*	NITE Biological Resource Center (NBRC, Tokyo, Japan)
*E. coli*	JM109	*relA supE44 endA1 hsdR17 gyrA96 mcrA mcrB*+ *thiΔ(lac-proAB)/F'(traD36 proAB*+ *lacIq lacZΔM15)*	Sambrook et al., 1989
**Plasmids**			
	pGFP7CA	*amyE gfp-H6, cat*	Imamura et al., 2011
	pCBGFPUV4	*gfp-cat*	Nishikawa and Kobayashi, 2021
	pCGEA8GA	*amyE*::P*_*cgeA*_*-*cgeA-gfp*-H6 *cat*	This work

**Table 2:** Primers used in this study.

**Primers**	**Sequence**	**Restriction site**
AMYE980	5′-ATGAAGCTTTCCGTTTAGGCTGGGCG-3′	*Hin*dIII
AMYE1860R	5′-TTTAAGCTTAGATCTGGTTGTATCCGTGTCCGC-3′	*Hin*dIII
CGEAM490	5′-CCAGGATCCAACACTTGAGAGTGAAACA-3′	*Bam*HI
CGEA398R	5′-GGACTCGAGGAAAAGAACGTAACGCTTTC-3′	*Xho*I
UV4-F	5′-ATCGGATCCGGCGGAGGCATGAG-3′	
cat-R	5′-AGTACAGTCGGCATTATCTC-3′	
cat-T-R	5′-AGTACAGTCGGCATTATCTCCAAAAAACCCCTCAAGACCCGTTTAGAGGCCCCAAGGGGTTATGCTAGTATTATAA	
	AAGCCAGTCATTAGGCCTATCTGAC-3′	
ATPC-F1	5′-CCGTTAAAGTCAATATCGTTACTCC-3′	
ATPC-R1	5′-CTCATGCCTCCGCCGGATCCGATTTTCCCTGCTACATCCAATC-3′	
ATPC-F2	5′-GAGATAATGCCGACTGTACTAATCAAACAGCGACAGCAACG-3′	
ATPC-R2	5′-AAAACTCGAGCTATTCGCTTCAACC-3′	
LONB-F1	5′-GCGACCACGAGAATGCCAAACG-3′	
LONB-R1	5′-CTCATGCCTCCGCCGGATCCGATAACGGATTCTTTATTGATTTCG-3′	
LONB-F2	5′-GAGATAATGCCGACTGTACTCATATCAGAAAGAAAGGGTATACTACG-3′	
LONB-R2	5′-GCGCTGTTTCTTTCACATGATCAGG-3′	
QOXB-F1	5′-TTGAGTTTCTTAGTTTGGACTCACC-3′	
QOXB-R1	5′-CTCATGCCTCCGCCGGATCCGATTTCGGAAATCTTTCTTTCCG-3′	
QOXB-F2	5′-GAGATAATGCCGACTGTACTTGAGTTATGGAACATGCAGAACACG-3′	
QOXB-R2	5′-TATTCGTTATGGCCTGAATGC-3′	

*B. subtilis* and other *Bacillales* species were grown in LB broth and Schaeffer's medium (Schaeffer et al., 1965). The conditions for the sporulation of *B. subtilis* have been described previously (Kuwana et al., 2002).

In addition to *B. subtilis*, the following *Bacillales* spore-forming bacteria were used: *Niallia circulans* NBRC 13629, *B. licheniformis* ATCC 14580, *Brevibacillus brevis* NBRC 100599, *Lysinibacillus sphaericus* NBRC 3526, and *Paenibacillus polymyxa* NBRC15309. The strains were obtained from the NITE Biological Resource Center (NBRC, Tokyo, Japan) and the American Type Culture Collection (ATCC, Manassas, VA, USA). Growth conditions were as follows: *B. licheniformis* ATCC 14580 was incubated on Schaeffer's agar medium at 24°C for 36 h. *N. circulans* NBRC 13629, *L. sphaericus* NBRC 3526, and *P. polymyxa* NBRC15309 were incubated on LB agar at 37°C for 24 h, followed by 25°C for additional 24 h to facilitate efficient spore release from mother cells.

Recombinant DNA preparations were carried out following standard protocols (Sambrook et al., 1989). Preparations of competent cells, transformation, and extraction of chromosomal DNA for *B. subtilis* were performed as described previously (Cutting and Vander Horn, 1990). When required, antibiotics were added at the following concentrations: chloramphenicol: 5 μg/mL and erythromycin: 0.5 μg/mL.

### 2.2 Phase contrast and fluorescence microscopy

Bacterial cells were cultured in LB or Schaeffer's medium, and aliquots were transferred to tubes for staining. Six fluorescence reagents were used in this study. Cells were stained with different combinations of the following dyes in 10 mM Tris-HCl (pH 7.6) at 25°C for 10 min as described previously (Kuwana et al., 2023): APBT (0.01 mg/mL; Tokyo Chemical Industry, Tokyo, Japan), which stains the cell membrane and/or cell wall (Kuwana et al., 2023); safranin O (0.01 mg/mL; Waldeck GmbH & Co. KG, Münster, Germany), which is commonly used as a counterstain in gram staining (Bartholomew and Mittwer, 1952) and also for measuring mitochondrial membrane potential due to its excitation/emission wavelengths (495/587 nm). In plant tissues, it binds to acidic lignin polymers, and its fluorescence peak shifts depending on lignin content (Baldacci-Cresp et al., 2020); thioflavin T (0.01 mg/mL; Sigma-Aldrich, Tokyo, Japan) is typically used to detect amyloid fibrils and to analyze the cell structure of *B. subtilis* (Kuwana et al., 2023). Following staining, cells were transferred to microscope slides for imaging.

Phase contrast and fluorescence microscopy images of *B. subtilis, B. licheniformis, N. circulans, B. brevis, L. sphaericus*, and *P. polymyxa* cells were acquired using an Olympus BX51 phase-contrast microscope equipped with additional fluorescence attachments and the following mirror cube units (Olympus, Tokyo, Japan): green fluorescence (U-MGFPHQ) for GFP, auramine O, safranin O, and thioflavin T; red fluorescence (U-MWG2) for congo red, rhodamine B, and safranin O; and blue fluorescence (U-MNUA2) for APBT. Imaging was performed using a UPlanApo 100X oil iris 3 pH objective lens and a U-TV1X-2 camera adapter (Olympus, Tokyo, Japan). Images were captured using an ORCA-SPARK digital CMOS camera C11440-36U (Hamamatsu Photonics Inc. Shizuoka, Japan) and analyzed using CellSens imaging software (Olympus, Tokyo, Japan). The exposure time for fluorescence imaging ranged from 0.25 s to 4.0 s. Contrast and tone balance were adjusted using CellSens. For each strain, the images were scaled to the same intensity range.

### 2.3 Measurement of spore cell length and fluorescence localization

Mature spores, characterized by high refractive brightness, exhibit a dark outer edge under phase-contrast microscopy. To determine the precise location of fluorescence within the spores, we identified the negative peak of phase-contrast intensity at the boundary between the spore's interior and exterior, following the protocol described by Imamura et al. (2010). Spore length was determined by measuring the distance between the negative peaks observed at both poles of the spore under a phase-contrast microscope. Measurements were performed using the CellSens imaging software (Olympus, Tokyo, Japan). Fluorescence microscopy of spore coat and cortex proteins fused with GFP showed a ring-shaped fluorescence pattern along the periphery of the spores. Based on the method of Imamura et al. (2010), fluorescence localization was quantified by identifying the positive fluorescence peaks of the GFP fusion protein at both poles of the spore's long axis. The distance between the two positive peaks was calculated using CellSens software. For each sample, 10 spores were analyzed, and we measured the following distances: negative peak distance (spore length from phase-contrast microscopy images) and positive peak distance (fluorescence intensity peaks from fluorescence microscopy images by fluorescent reagents and/or GFP fusion proteins). The relative fluorescence localization in spores was computed as the average of these measured distances.

### 2.4 Statistical analyses

To determine statistical significance, we used a two-way analysis of variance (ANOVA) followed by Tukey's multiple comparison test to compare the means of multiple groups and identify significant differences. Results with *P* ≤ 0.05 were considered statistically significant, and all statistical analyses were performed using Microsoft Excel.

## 3 Results

### 3.1 Comparison of APBT and safranin O staining and the diversity among *Bacillales* spores

**Figure 1:**
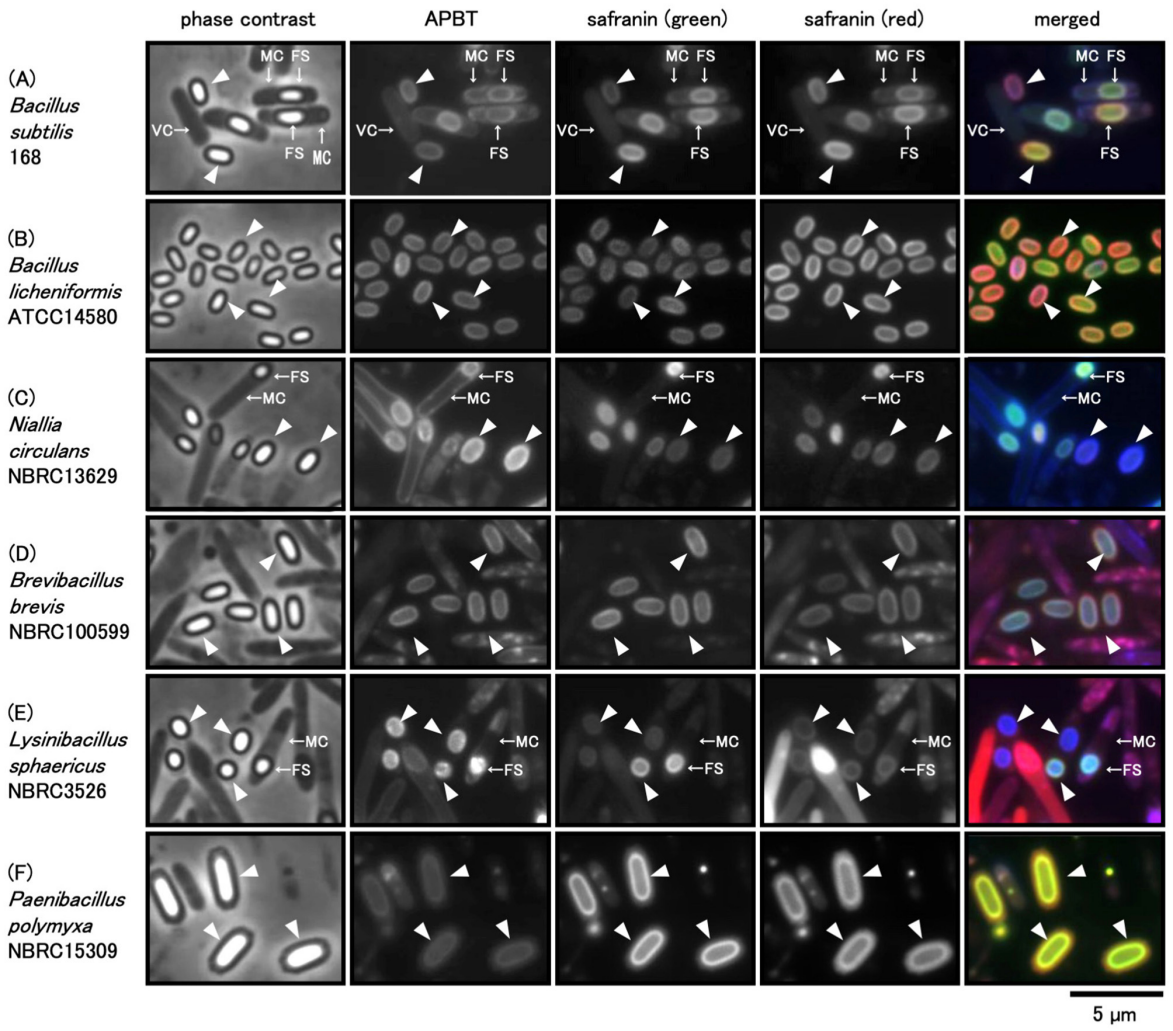
Phase contrast and fluorescence microscopic images of *Bacillales* spores stained with APBT and safranin O. cells of *Bacillus subtilis* **(A)**, *B. licheniformis* **(B)***, Niallia circulans* **(C)***, Brevibacillus brevis* **(D)***, Lysinibacillus sphaericus* **(E)**, and *Paenibacillus polymyxa* **(F)** were suspended in a mixture of 2-(4-aminophenyl) benzothiazole (APBT) and safranin O and observed using phase contrast and fluorescence microscopy. From left to right: phase contrast images, APBT fluorescence, green fluorescence of safranin O, red fluorescence of safranin O, and merged images. Blue represents APBT fluorescence, while green and red indicate the green and red fluorescence of safranin O, respectively. Arrows indicate vegetative cells (VCs), mother cells (MCs), and forespores (FSs), and arrowheads indicate mature spores (MSs) in phase-contrast microscopy images. The scale bar represents 5 μm.

*B. subtilis* 168, *B. licheniformis* ATCC 14580, *N. circulans* NBRC 13629, *B. brevis* NBRC 100599, *L. sphaericus* NBRC 3526, and *P. polymyxa* NBRC 15309, all belonging to *Bacillales*, were grown on the Schaeffer's or LB plates at 37°C (see Methods). Harvested cells were stained with a mixture of APBT and safranin O and observed under a phase-contrast and fluorescence microscope, identifying oval-shaped mature spores for most species with notable variations in size and shape under phase-contrast microscopy (Figure 1). *L. sphaericus* NBRC 3526 spores were the smallest, while those of *P. polymyxa* NBRC 15309 were the largest among the six species. *L. sphaericus* NBRC3526 spores exhibited a mix of circular and oval shapes with variations in size. APBT fluorescence was detected along the periphery of vegetative cells, mother cells, forespores, and mature spores in all strains (Figure 1). This finding is comparable with previous reports, which suggested that APBT stains the cell membrane (Kuwana et al., 2023, 2024). Safranin O fluorescence was detected using green and red mirror cube units (see Methods). Though both green and red fluorescence signals of safranin O were detected in all cell types, the intensity was stronger in the forespores and mature spores than in the vegetative and mother cells (Figure 1). Additionally, safranin O fluorescence was detected only at the periphery of the forespores or mature spores and not in the core of the spores. The green fluorescence of safranin O was similar to that of APBT in *B. subtilis* cells. The red fluorescence of safranin O was detected in the mother cells of *B. brevis* NBRC 100599 and *L. sphaericus* NBRC 3526, and the cores of some forespores of *N. circulans* NBRC 13629 and *L. sphaericus* NBRC 3526.

To evaluate whether the observed fluorescence signals were due to intrinsic autofluorescence of the spores, we examined unstained *B. subtilis* 168 spores using the same filter sets and a range of exposure times. Autofluorescence signals were only detectable with exposure times of >1 s for blue and > 8 s for green and red filters (Supplementary Figure S1). Fluorescence microscopy experiments were therefore conducted with exposure times below these thresholds. Similar background levels were confirmed in other *Bacillales* species. These findings indicate that the observed fluorescence signal primarily results from the added fluorescent dyes rather than intrinsic autofluorescence.

We performed a supplemental experiment to assess whether safranin O alone could visualize spores by brightfield microscopy. As shown in Supplementary Figure S2, vegetative and mother cells were stained with higher concentrations of safranin O (2.5 and 1 mg/mL), whereas the mature spores did not stain clearly. In contrast, no cells were visibly stained at a lower concentration (0.01 mg/mL).

**Figure 2:**
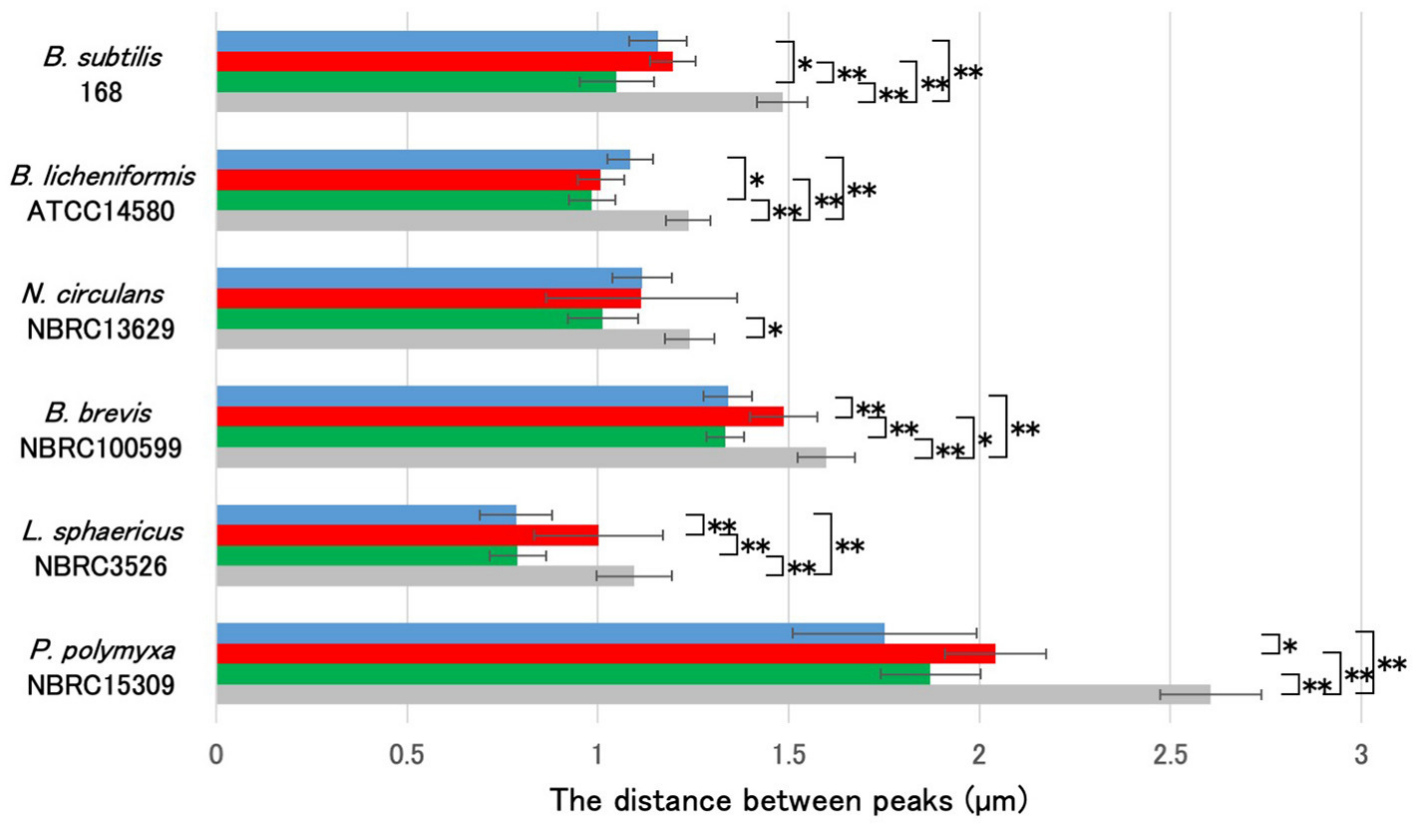
Comparative analysis of cell length and fluorescence peak distances in *Bacillales* species spores stained with APBT and Safranin O. Using the image analysis software CellSens, the distances between the negative peaks in the phase-contrast microscopy images and between the positive peaks in the fluorescence microscopy images were measured for 10 spores each. Bar colors represent: blue, APBT fluorescence; green, safranin O green channel; red, safranin O red channel; gray, phase-contrast measurements. The average values (μm) are shown in the graph. Data are presented as mean ± SD calculated. Asterisks indicate significant differences determined via two-way ANOVA, followed by Tukey's multiple comparison test to compare the conditions. *P < 0.05, **P < 0.01. APBT, 2-(4-aminophenyl) benzothiazole; SD, standard deviation; ANOVA, analysis of variance.

Ten spores with similar morphology were selected from each sample for analysis. We measured the distance between the negative peaks along the long axis of the spores in the phase-contrast microscopy images, and the distance between the positive fluorescence signal peaks from the applied fluorescent reagents (Figure 2). We observed that the average length of the long axis of *L. sphaericus* NBRC 3526 spores was the smallest, and that of *P. polymyxa* NBRC15309 was the largest, with a more than two-fold size difference. In mature spores of *B. subtilis* 168, *B. licheniformis* ATCC 14580, *N. circulans* NBRC 13629, *B. brevis* NBRC 100599, *L. sphaericus* NBRC 3526, and *P. polymyxa* NBRC 15309, the distance between the positive peaks of APBT, green safranin O and red safranin O fluorescence was smaller than the length of the long axis of each mature spore. These results revealed that all the fluorescent reagents selectively stained specific structures of *Bacillales* mature spores.

Phase contrast microscopy results of the distance between the negative peaks indicated that *B. subtilis* 168 and *B. brevis* NBRC 100599 spores had similar sizes; *P. polymyxa* NBRC 15309 spores were the largest, while the spore size of *L. sphaericus* NBRC 3526 was the smallest. Consequently, despite variations in spore size, we observed that the distance between the positive peaks of red fluorescence from safranin O consistently exceeded that of the positive peaks from the red fluorescence of APBT and the green fluorescence of safranin O across these four strains. The fluorescence of APBT and safranin O in *B. licheniformis* ATCC 14580 and *N. circulans* NBRC 13629 spores were unique. In *B. licheniformis* ATCC 14580 and *N. circulans* NBRC 13629 spores, fluorescence was detected primarily at the spore periphery, but their staining profiles differed. In *B. licheniformis*, APBT fluorescence peaks were spaced greater than those of green safranin O, whereas red safranin O matched APBT. In contrast, in *N. circulans*, all three dyes produced similar peak distances. These findings highlight species-specific variations in spore size and staining patterns, reflecting structural diversity within *Bacillales* spores.

### 3.2 Comparison of APBT fluorescence and GFP-labeled spore proteins

**Figure 3:**
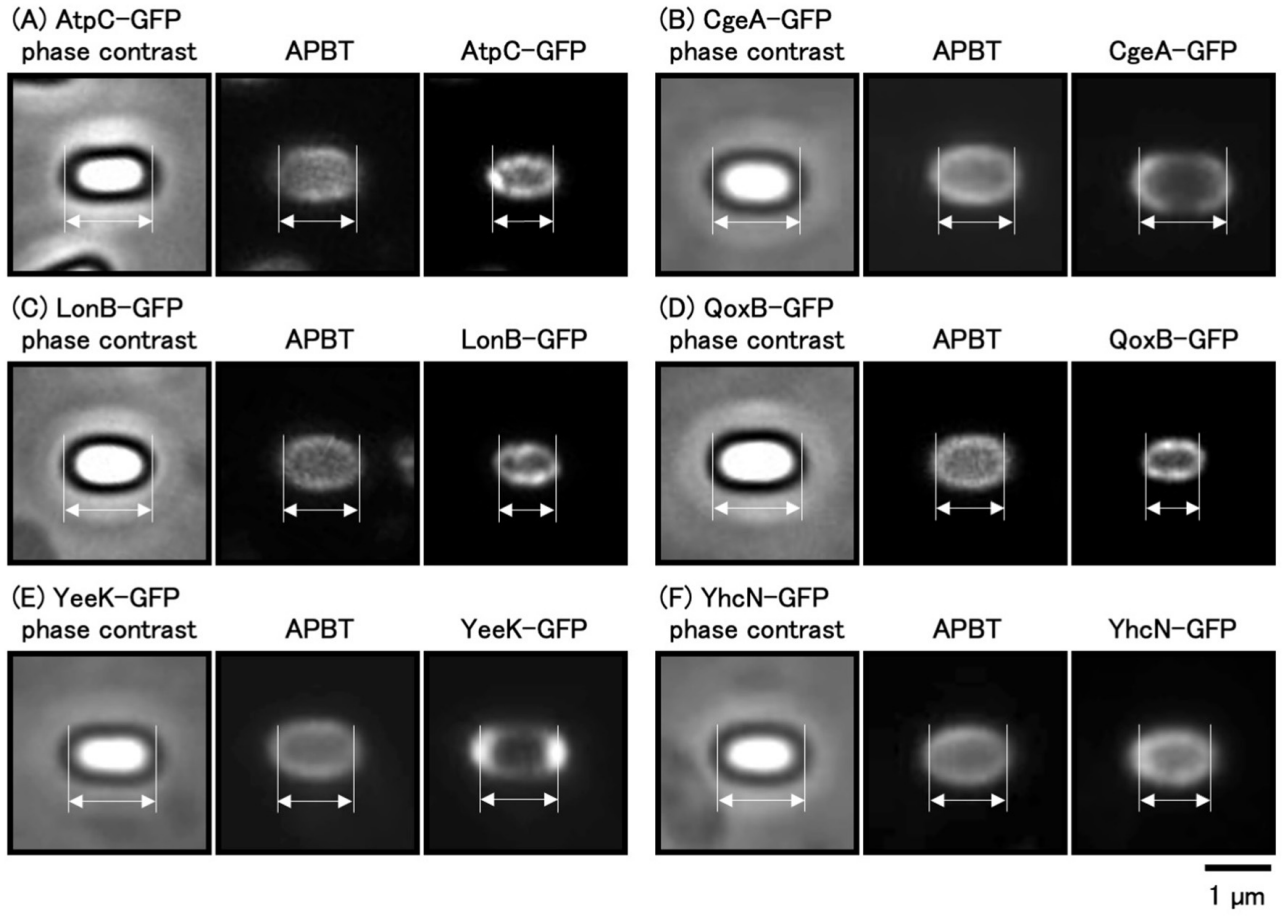
Fluorescence images of APBT and GFP in GFP fusion strains of *Bacillus subtilis. B. subtilis* strains harboring *atpC-gfp* **(A)**, *cgeA-gfp* **(B)**, *lonB-gfp* **(C)**, *qoxB-gfp* **(D)**, *yeeK-gfp* **(E)**, and *yhcN-gfp* **(F)** were stained with APBT and observed using phase contrast and fluorescence microscopy. From left to right: phase contrast, APBT fluorescence, and GFP images. The arrows with opposite directions indicate the distance between the negative peaks in the phase contrast images and between the positive peaks in the fluorescence images. Scale bar: 1 μm. APBT, 2-(4-aminophenyl) benzothiazole; GFP, green fluorescence protein.

**Figure 4:**
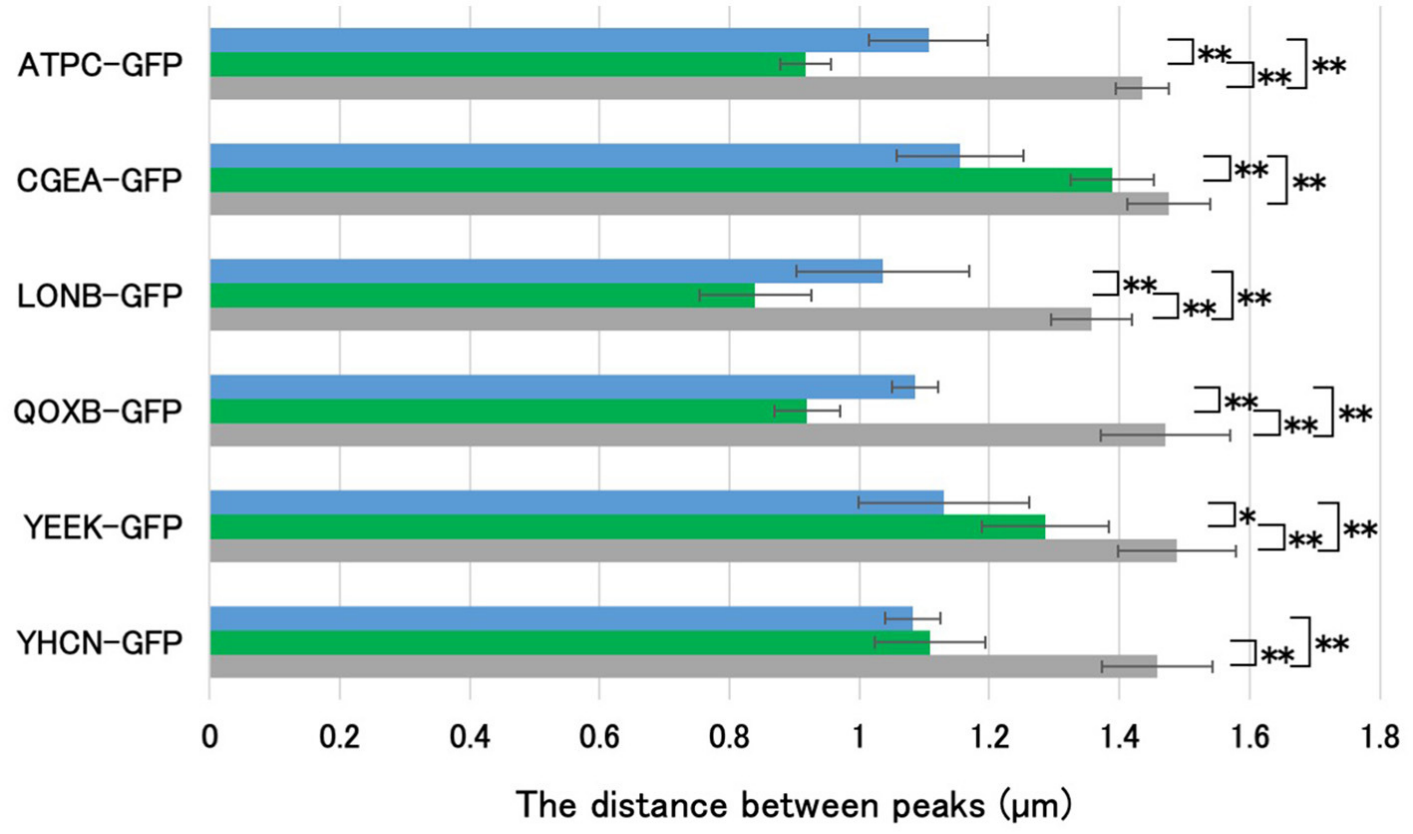
Determination of the staining region of APBT using GFP-fused spore proteins. Using the image analysis software CellSens, the distance between the negative peaks in the phase-contrast microscopy images and between the positive peaks in the fluorescence microscopy images were measured for 10 spores each, as shown in Figure 3. Bar colors represent: blue, APBT fluorescence; green, GFP signal; gray, phase-contrast measurements. The average values (μm) are shown in the graph. Data are presented as mean ± SD. Asterisks indicate significant differences determined via two-way ANOVA, followed by Tukey's multiple comparison tests to compare the conditions. *P < 0.05, **P < 0.01. APBT, 2-(4-aminophenyl) benzothiazole; GFP, green fluorescence protein; SD, standard deviation; ANOVA, analysis of variance.

In mature *B. subtilis* spores, GFP fusion strains of spore proteins were used to compare the detectable fluorescence of GFP with that of APBT. We previously reported the localization of spore proteins using GFP fusion strains (Imamura et al., 2010, 2011). CgeA-GFP is located in the crust, YeeK-GFP is located in the inner spore coat, and YhcN-GFP is located in the cortex and inner spore membrane (Takamatsu et al., 2009; Imamura et al., 2010; Zheng et al., 2016; Liu et al., 2022). To determine the proportion of APBT staining in the spores, we used CgeA-GFP, YeeK-GFP, and YhcN-GFP strains as marker proteins for the crust, inner coat, and cortex, respectively. The *atpC* and *qoxB* genes encode components of the ATP synthase and cytochrome aa3 quinol oxidase (subunit I), respectively, and AtpC and QoxB localize to the cell membrane (Meile et al., 2006; Hahne et al., 2008). The *lonB* gene encodes a Lon-like ATP-dependent protease, whose expression is dependent on *sigF* during early sporulation; LonB localizes to the inner spore membrane (Serrano et al., 2001; Simmons et al., 2008). We constructed the GFP fusion strains AtpC-GFP, LonB-GFP, and QoxB-GFP to mark the inner spore membrane. The GFP fusion strains were grown on Schaeffer's plates at 37°C for 24 h, after which the cells were collected and stained with APBT and observed under phase-contrast and fluorescence microscopy (Figure 3). We measured the distance between the negative peaks of the mature spores in the phase-contrast images to define the length of the long axis of the mature spore. We also measured the distance between the positive APBT and GFP fluorescence peaks in fluorescence images (Figures 3, 4). We found that the average distance between the positive peaks of APBT and GFP fluorescence was smaller than the length of the long axis of mature spores. The distance between the positive peaks of APBT fluorescence of APBT was also smaller than that of CgeA-GFP and YeeK-GFP (Figures 3B–E, 4) and closer to that of YhcN-GFP (Figures 3F, 4). Previous studies have reported that YhcN localizes in the cortex and inner spore membrane (Takamatsu et al., 2009; Imamura et al., 2010; Liu et al., 2022). We compared the fluorescence of these GFP strains with that of APBT (Figures 3A, C, D, 4). The fluorescence of AtpC-GFP, LonB-GFP, and QoxB-GFP were detected in the inner spore membrane. The distance between the positive peaks of APBT fluorescence peaks was larger than that of AtpC-GFP, LonB-GFP, and QoxB-GFP. Based on these results, we concluded that APBT stains around the inner portion of the inner spore coat and the outer portion of the inner spore membrane, similar to YhcN-GFP.

### 3.3 Comparison of fluorescent reagents staining in spores

**Figure 5:**
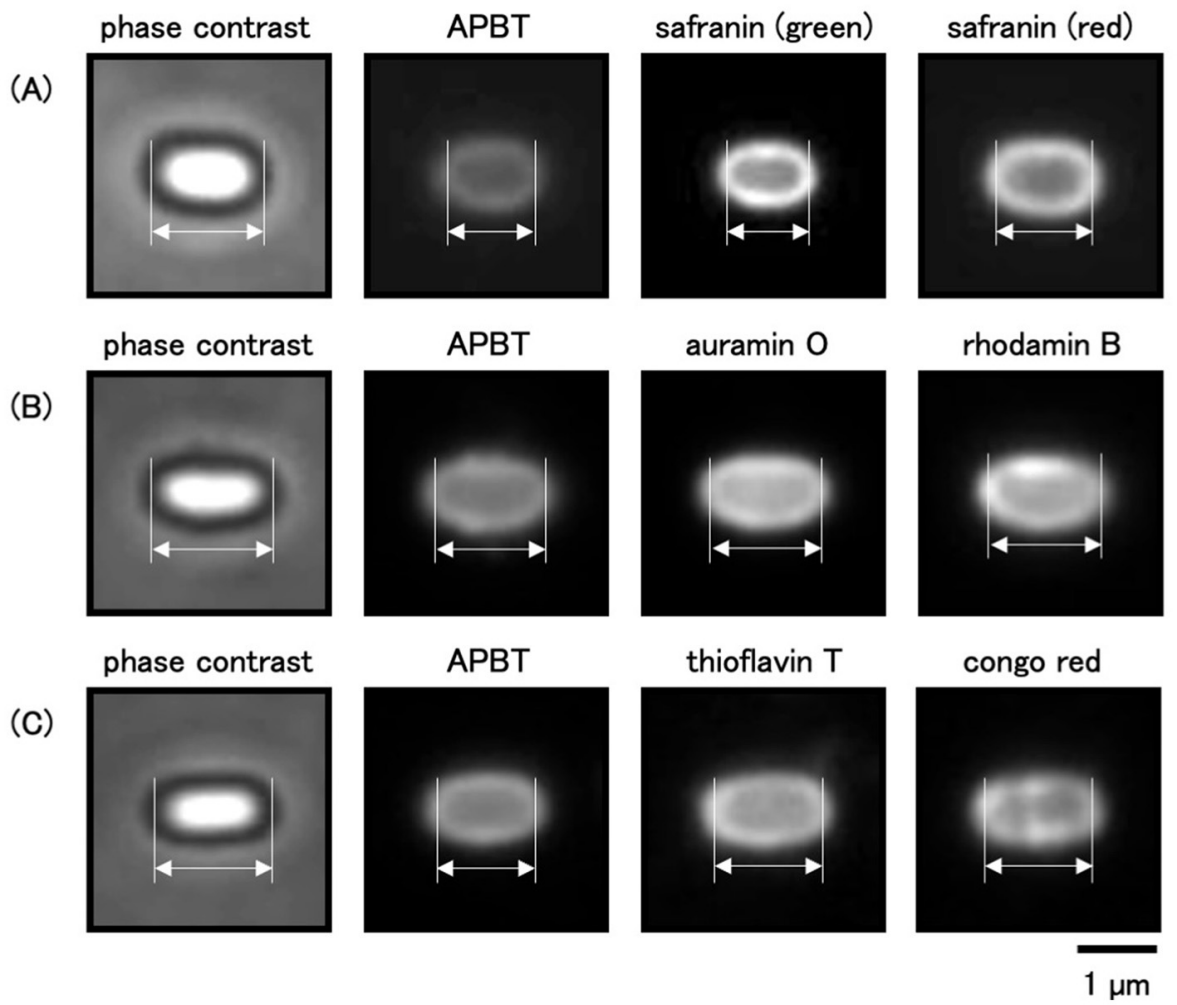
Comparative analysis of staining regions in spores with multiple fluorescent reagents. *Bacillus subtilis* 168 cells were suspended in the mixture of APBT and safranin O **(A)**, APBT, auramine O, and rhodamine B **(B)**, or APBT, thioflavin T, and congo red **(C)** and observed using the phase-contrast and fluorescence microscopy. From left to right: phase contrast, APBT fluorescence, green fluorescence, and red fluorescence images. The arrows with opposite directions indicate the distance between the negative peaks in the phase contrast images and the positive peaks in fluorescence images. Scale bar: 1 μm. APBT, 2-(4-aminophenyl) benzothiazole.

**Figure 6:**
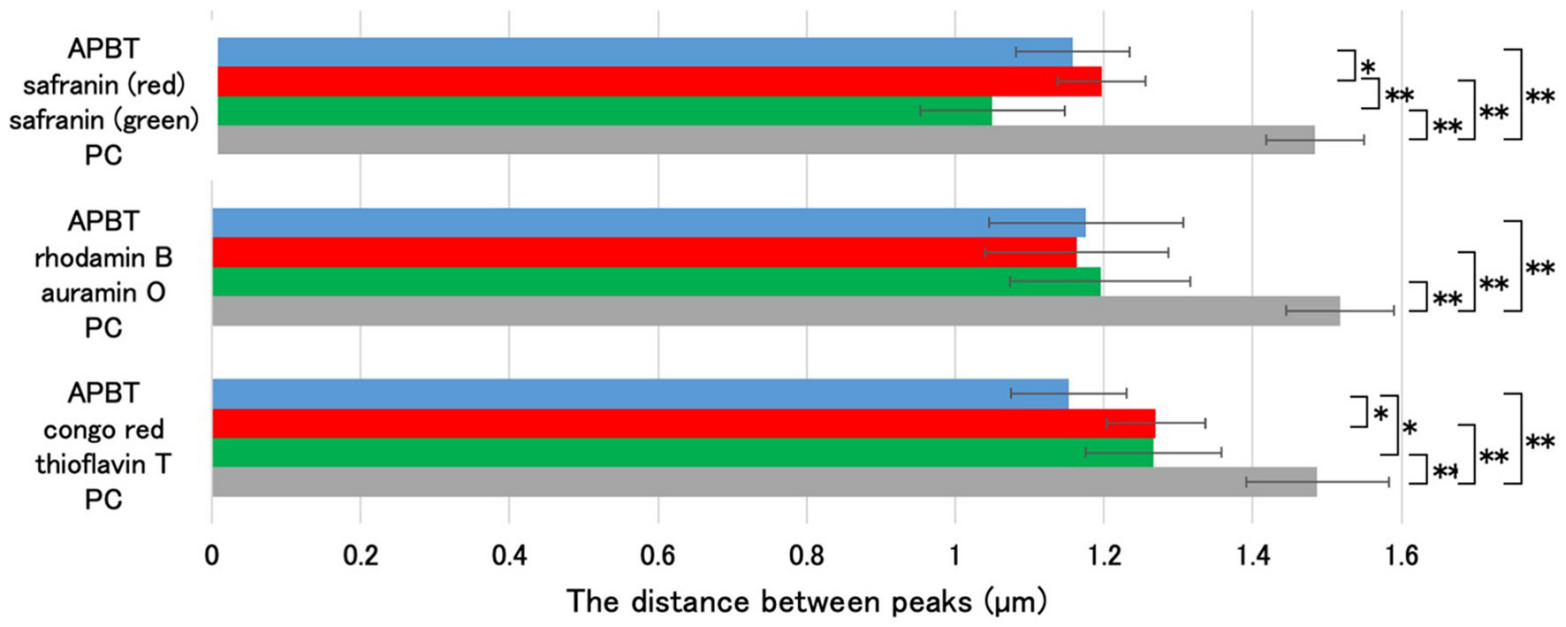
Determination of the staining region of safranin O using multiple fluorescent reagents. Using the image analysis software CellSens, the distance between the negative peaks in the phase-contrast microscopy images and the positive peaks in the fluorescence microscopy images were measured for 10 spores each, as shown in Figure 5. The average values (μm) are shown in the graph. Data are presented as mean ± SD calculated. Asterisks indicate significant differences determined via two-way ANOVA, followed by Tukey's multiple comparison test to compare the conditions. *P < 0.05, **P < 0.01. SD, standard deviation; ANOVA, analysis of variance.

Fluorescent reagents such as auramine O, rhodamine B, thioflavin T, and congo red have been used to analyze spore structures (Kuwana et al., 2023, 2024). In this study, we determined the regions of mature spores stained by these fluorescent reagents with APBT fluorescence as a reference (Figure 5). *B. subtilis* 168 cells were grown on Schaeffer's plates at 37°C for 24 h, collected, and stained with a mixture of fluorescent reagents. We then observed the cells using phase contrast and fluorescence microscopy (Figure 5). We measured the distance between the negative peaks of the mature spores in the phase-contrast images to define the length of the long axis of the mature spore. We also measured the distance between the positive fluorescence peaks of the reagents in the fluorescence images (Figure 6). The distance between the positive fluorescence peaks of APBT, green safranin O, red safranin O, auramine O, rhodamine B, thioflavin T, and congo red were all shorter than the length of the long axis of the mature spore. These results indicated these fluorescent reagents stained specific structures in *B. subtilis* mature spores. The distance between the red fluorescence peaks of safranin O was greater than that of APBT, whereas the distance between the green fluorescence peaks of safranin O was slightly smaller than that of APBT, although the difference was not significant. The distance between the positive rhodamine B and auramine O fluorescence peaks was similar to that of the positive APBT fluorescence peaks. These results show that safranin O stains different regions of *B. subtilis* spores with red and green fluorescence.

### 3.4 Effect of sporulation-related gene mutations on safranin O staining

**Figure 7:**
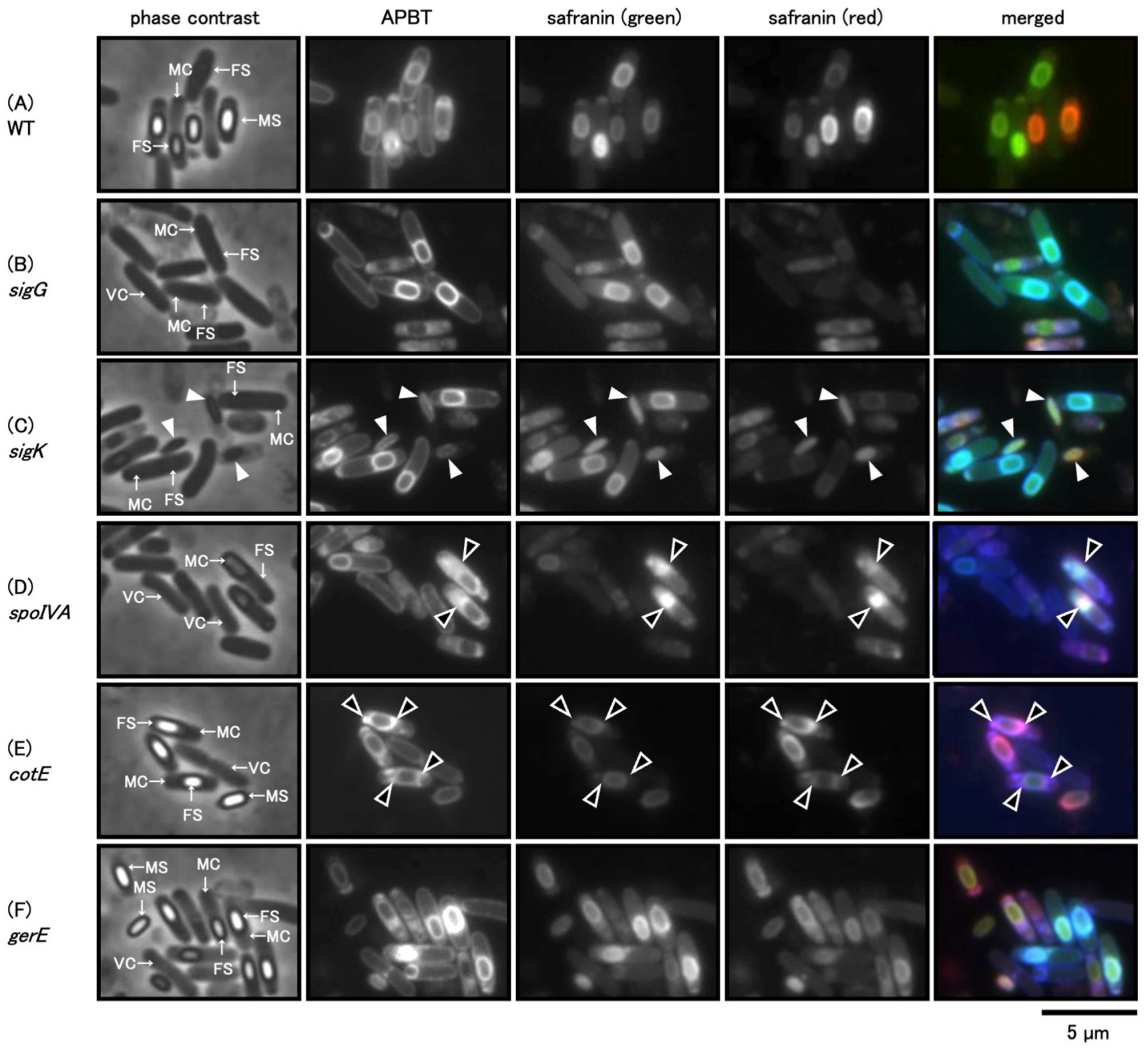
Phase-contrast and fluorescence microscopy images of *Bacillus subtilis* sporulation-deficient cells stained with APBT and Safranin O. *B. subtilis* wild-type **(A)**, *sigG* **(B)**, *sigK* **(C)**, *spoIVA* **(D)**, *cotE* **(E)**, and *gerE* **(F)** gene-deficient strains were suspended in the mixture of APBT and safranin O and observed using the phase-contrast and fluorescence microscopy; blue indicates APBT fluorescence, while green and red represent the green and red fluorescence of safranin O, respectively. From left to right: phase contrast, APBT fluorescence, green fluorescence of safranin O, red fluorescence of safranin O, and merged images. Arrows in phase-contrast images indicate vegetative cells (VCs), mother cells (MCs), and forespores (FSs). White arrowheads indicate abnormal spores of the *sigK* gene-deficient cells, and black arrowheads indicate abnormal cortex or spore coat structures in the *spoIVA* or *cotE* gene-deficient cells. The scale bar represents 5 μm. APBT, 2-(4-aminophenyl) benzothiazole.

To analyze how sporulation influences safranin O staining, we used wild-type, *sigG, sigK*, and *spoIVA* mutant strains deficient in the development of the cortex and spore coat (Driks, 1999; McKenney et al., 2013). We also used *cotE* and *gerE* mutant strains that are deficient in inner and outer spore coat development, respectively (Driks, 1999; McKenney et al., 2013). The mutant strains were grown on Schaeffer's plates at 37°C for 24 h. Cells were collected and stained with a mixture of APBT and safranin O and observed using phase contrast and fluorescence microscopy (Figure 7). No phase-bright forespores were detected in the *sigG, sigK*, and *spoIVA* mutant strains. However, APBT fluorescence revealed the engulfed forespore, which allowed for a distinction between the mother and vegetative cells (Figures 7B–D). Safranin O green fluorescence was detected at the periphery of the forespore in *sigG* and *sigK* mutant strain cells, similar to wild-type cells and APBT. In contrast, the red fluorescence of safranin O was less distinct in the *sigG* and *sigK* mutant strain cells than in the wild-type cells (Figures 7A–C). In *sigK* mutants, green and red safranin O fluorescence appeared as an oval structure within lysed mother cells, co-localizing with APBT fluorescence (Figure 7C, white arrowheads). A previous study showed an abnormal mother cell structure in *spoIVA* mutants (Roels et al., 1992). The APBT fluorescence was detected at the periphery of the *spoIVA* vegetative cell, mother cell, and forespore, and it was especially strong at the abnormal structure in the mother cell (Figure 7D, black arrowheads). The green and red fluorescence of safranin O was hardly detected at the periphery of the *spoIVA* forespore but was strongly detected in the abnormal structure of the *spoIVA* mother cells (Figure 7D, black arrowheads).

We observed the phase-bright forespores and mature spores in the *cotE* and *gerE* mutant cells (Figures 7E, F). We could distinguish the vegetative cell, mother cell, forespore, and mature spore via APBT fluorescence. Green and red safranin O fluorescence was detected at the periphery of the forespore in *cotE* mutant cells, with the green fluorescence being weaker than the red fluorescence. Additionally, both APBT and red safranin O fluorescence were detected in some abnormal structures as strong spots in *cotE* mutant cells (Figure 7E, black arrowheads). In contrast, the APBT and safranin O fluorescence staining pattern in *gerE* mutant cells was similar to that in wild-type cells (Figures 7A, F). We concluded that coat and cortex development influenced safranin O staining in sporulating cells.

## 4 Discussion

Safranin O is commonly used in gram staining for bacterial identification. However, in conventional Gram staining, bacterial endospores are often poorly stained and appear as clear or “ghost-like” areas under conventional light microscopy due to the thick and impermeable spore coat. To overcome this limitation, specialized staining protocols such as the Schaeffer-Fulton method are typically employed (Schaeffer and Fulton, 1933). These methods rely on heat or acid treatment to allow penetration of primary stains like malachite green, followed by safranin O to counterstain vegetative or mother cells. However, these treatments are incompatible with live-cell imaging and may alter or interfere with the structural integrity of spores. To evaluate whether safranin O alone could be used for spore visualization without such treatments, we compared its performance under light and fluorescence microscopy, as shown in Supplementary Figure S2. The results in Supplementary Figure S2 indicate that safranin O is ineffective for spore visualization by conventional light microscopy without additional treatments such as heat fixation. In contrast, under fluorescence microscopy, even low concentrations of safranin O yielded distinct peripheral staining of forespores and mature spores without needing fixation or harsh treatments. These findings support the use of our non-destructive fluorescence protocol for live-compatible visualization of spore structures, making it better suited for dynamic or physiological studies of sporulation.

Previous studies have shown that dormant *B. subtilis* spores exhibit intrinsic autofluorescence attributed to components of the spore coat (Magge et al., 2009). In particular, autofluorescence is substantially reduced in *cotE gerE* double mutants lacking major coat proteins. In the present study, we also examined the spores from *cotE* and *gerE* mutants; however, fluorescence signals were still detectable following dye staining (Figure 7), suggesting that the observed fluorescence originates from dye interaction rather than intrinsic autofluorescence alone.

To further distinguish dye-derived fluorescence from background autofluorescence, we carefully optimized exposure times during imaging. As shown in Supplementary Figure S1, autofluorescence signals were detectable at long exposure times (≥1 s for blue, ≥8 s for green/red), whereas our staining images were acquired at shorter exposure times. These findings indicate that the fluorescence observed in our experiments is not due to autofluorescence, but rather due to the binding of fluorescent dyes to specific spore structures. These findings emphasize the importance of controlling for autofluorescence when using fluorescence microscopy to visualize bacterial spores. Our approach provides a non-destructive and live-cell compatible alternative to traditional staining methods for studying spore architecture and dynamics.

Although the red and green fluorescence of safranin O has been utilized in plant tissue observation studies, its use for bacterial observation has not yet been reported (Baldacci-Cresp et al., 2020). In this study, we demonstrated that spore-forming bacteria, such as *B. subtilis* 168, *B. licheniformis* ATCC 14580, *N. circulans* NBRC 13629, *B. brevis* NBRC 100599, *L. sphaericus* NBRC 3526, and *P. polymyxa* NBRC 15309, produce green and red fluorescence when stained with safranin O. This fluorescence facilitated structural analysis of spores.

TEM studies have shown that the spores of bacteria belonging to the phylum *Bacillota* have a conserved structure consisting of a core, inner spore membrane, cortex, outer spore membrane, spore coat, crust, and exosporium (Driks and Eichenberger, 2016; Galperin et al., 2022). In our present APBT and safranin O fluorescence experiments, we observed blue, green, and red fluorescence in spores of all six spore-forming bacterial species studied, with distinct morphological differences in fluorescence intensity, average length, and stained area of each fluorescent dye (Figures 1, 2). This result indicates that the spores of the phylum *Bacillota* have diverse structures. APBT mainly stains the cell membrane, a common feature of these six bacterial species. The green and red fluorescence of safranin O was stronger in the forespores and mature spores than in the vegetative or mother cells (Figure 1). We demonstrated that fluorescent microscopy can be used to analyze cell morphology in living cells, with some safranin O staining sites being similar and others different among six species' spores. Next, we used GFP fusion and gene mutant strains of *B. subtilis* to determine the safranin O staining sites using APBT as a reference marker.

While fluorescence microscopy provides valuable fluorescence localization data, its spatial resolution is limited to ~300 nm due to the diffraction limit of light. This can restrict the ability to distinguish fine structural differences within bacterial spore layers. Although we did not have access to high-resolution imaging systems such as confocal or super-resolution microscopy in this study, we addressed this limitation by integrating quantitative fluorescence signal analysis with GFP-fusion proteins and specific fluorescent dyes. Previous studies have shown that structural insights can be achieved by combining fluorescence microscopy with statistical spatial analysis (Imamura et al., 2010; McKenney et al., 2010, 2013). Following this approach, we measured fluorescence intensity peak distances for fluorophores and compared them across multiple *Bacillales* species (Figure 2). These reproducible and statistically significant differences in localization patterns support the utility of our method in characterizing spore structures, even within the resolution limits of conventional fluorescence microscopy.

The long axis length of the spores was determined by measuring the distance between the negative peaks of the spores using phase-contrast microscopy (Imamura et al., 2010). The localization of the coat and cortex proteins of *B. subtilis* spores was determined by measuring the distance between the two positive fluorescence peaks of each GFP fusion protein (Imamura et al., 2010). The distance between the positive fluorescence peaks of GFP fusions of proteins such as CgeA-GFP, a marker of the outermost crust, closely matched the length of the *B. subtilis* spores (Figure 3B; Imamura et al., 2011). Furthermore, YeeK-GFP was used as an indicator of the inner coat; YhcN-GFP for the cortex and/or inner spore membrane; and AtpC-GFP, LonB-GFP, and QoxB-GFP for the inner spore membrane (Takamatsu et al., 2009; Imamura et al., 2010; Liu et al., 2022; Meile et al., 2006; Hahne et al., 2008; Serrano et al., 2001; Simmons et al., 2008). APBT stains the cell membranes of *B. subtilis* and *C. sporogenes* cells (Kuwana et al., 2023, 2024). In this study, we hypothesized that the distance between the positive fluorescent peaks of different staining reagents could identify the staining sites of the spores similar to GFP fusion proteins. Using APBT as a reference marker, we compared its blue fluorescence with the green fluorescence of GFP fusion proteins. The fluorescence of APBT was detected inside the fluorescence of YeeK-GFP (inner coat) but outside the fluorescence of AtpC-GFP, LonB-GFP, and QoxB-GFP (inner spore membrane) and was in close proximity to the fluorescence of YhcN-GFP (Figures 3, 4). These results suggest that APBT primarily stains near the outer spore membrane where the spore coat basement layer is formed. We hypothesize that the fluorescence of APBT detected outside AtpC-GFP, LonB-GFP, and QoxB-GFP may be due to its low permeability in spores. This is likely due to the complex structure of the spores and their resistance to various chemicals (Setlow, 2014).

In this study, *B. subtilis* spores were stained with multiple fluorescent reagents, such as APBT, safranin O, auramine O, rhodamine B, congo red, and thioflavin T, and their staining sites were analyzed using APBT as the reference marker (Figure 5). For all fluorescent reagents used in this study, the distance between the positive fluorescence peaks in *B. subtilis* spores was shorter than the total length (Figures 5, 6), suggesting that these reagents primarily stain internal structures rather than the crust. The distance between the positive peaks of red safranin O, congo red, and thioflavin T fluorescence was greater than that of APBT, whereas those for green safranin O, rhodamine B, and auramine O were similar to that of APBT. This finding indicates that the fluorescence of red safranin O, congo red, and thioflavin T was detected outside the APBT-stained regions, while the fluorescence of green safranin O, rhodamine B, and auramine O was detected inside the APBT-stained regions in *B. subtilis* spores.

We could not directly analyze the distance between the positive fluorescence peaks of green safranin O and the GFP fusion proteins of inner spore membrane proteins, such as AtpC, LonB, and QoxB. Therefore, we compared the distances between each measured positive peak and the corresponding cell sizes (Figures 4, 6). The average distances between the positive fluorescence peaks of AtpC-GFP, LonB-GFP, and QoxB-GFP were 0.91, 0.83, and 0.92 μm, respectively. In contrast, the average distance between the positive fluorescence peaks for green safranin O was 1.05 μm. These results suggest that the fluorescence of green safranin O was detected in a region similar to that of APBT on the outer side of the inner spore membrane.

**Figure 8:**
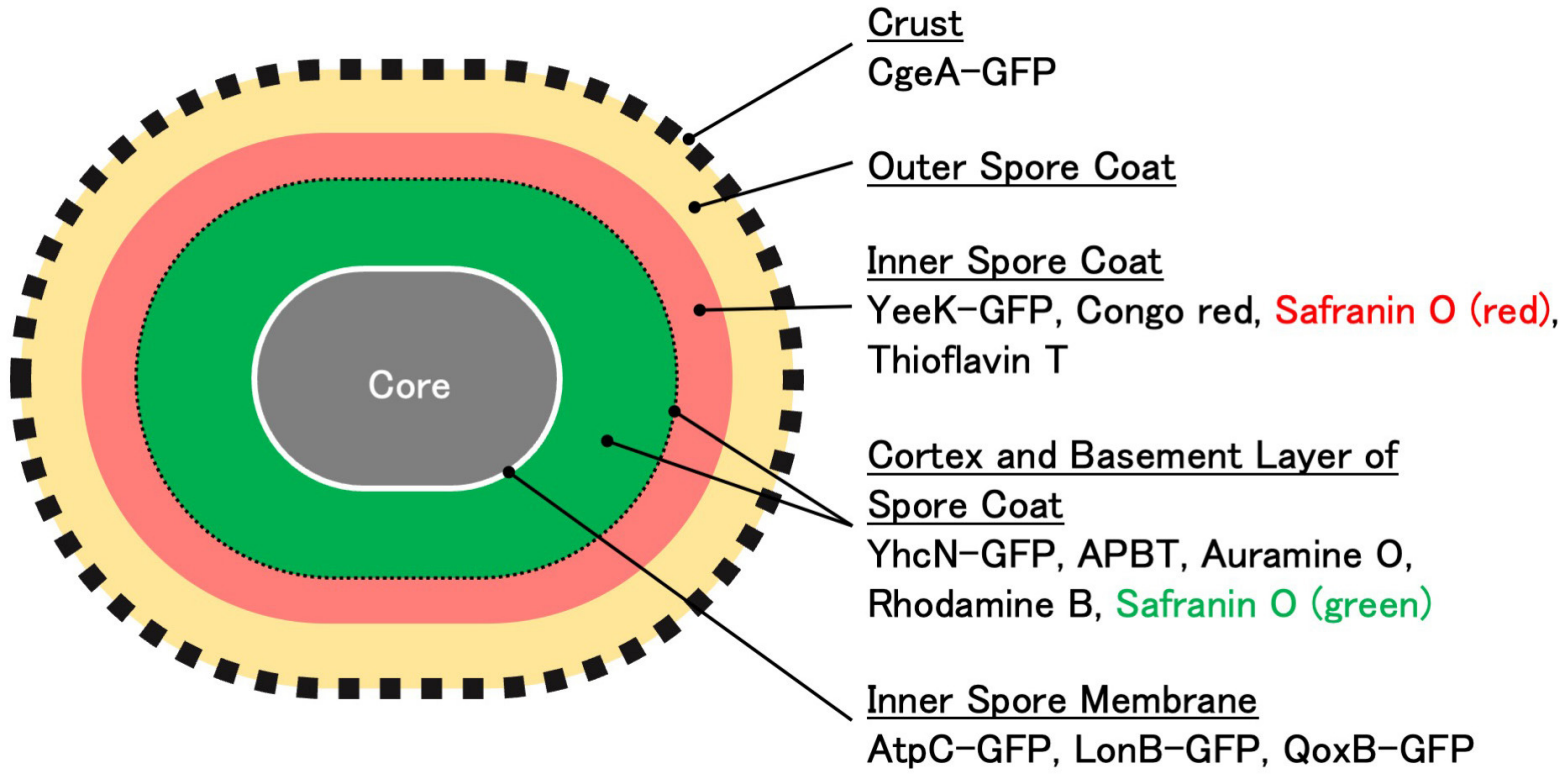
Illustration of the localization of GFP fusion proteins and the staining regions of fluorescent reagents in *Bacillus subtilis* spores A model of the cell structure of *B. subtilis* spores is presented. The localization sites of each GFP fusion protein and the staining regions of the fluorescent reagents were estimated by measuring the mean distance between the negative peaks in phase-contrast microscopy images and the mean distance between the positive peaks in fluorescence microscopy images.

APBT fluorescence was detected at the periphery of vegetative cells, mother cells, forespores, and mature spores in the sporulation gene-deficient mutants used in this study (Figure 7). This indicates that APBT stained the cell membrane. In contrast, defects in sporulation-specific genes affected the green and red fluorescence of safranin O (Figure 7). The red fluorescence of safranin O in the forespore was reduced in both the *sigG* and the s*igK* deficient cells, which are involved in the regulation of gene expression during the middle to late stages of sporulation as well as the development of the cortex and spore coat, respectively, compared to wild-type strains (Figures 7A–C). APBT and safranin O fluorescence were detected in the abnormal spores of *sigK* gene-deficient cells. In *spoIVA* mutant cells, which are involved in cortex and spore coat formation, the green and red fluorescence of safranin O was condensed in the mother cell (Figure 7D, black arrowheads). Similar results were observed in *cotE* mutant cells, which are mainly involved in inner spore coat formation (Figure 7E, black arrowheads). These results confirmed that the cortex and spore coat are the main staining sites for safranin O (green and red). In the *gerE* mutant cells, which are mainly involved in the gene expression of outer spore coat proteins, both the red and green fluorescence of safranin O in the forespore were similar (Figure 7F). This suggests that the red fluorescence of safranin O represents the inner and internal regions of the outer spore coat. None of the fluorescent reagents used in this study stained the same sites as the crustal structural protein CgeA-GFP. Based on these results, the main staining sites for the fluorescent reagents used in this study are shown in Figure 8.

Safranin O is commonly used for the fluorescent staining of plant tissues because it exhibits green and red fluorescence when it binds to acidic lignin polymers. In plant tissues, safranin O emits red fluorescence when the cell wall lignin content is high, while the emitted fluorescence is green when the cell wall lignin content is low (Baldacci-Cresp et al., 2020). Because bacterial spores do not contain lignin, the fluorescence shift observed in this study is likely due to other factors. Although we could not identify the specific substances stained by safranin O, we demonstrated its utility as a valuable tool for visualizing differences in spore structures.

Several fluorescent reagents have been used for the morphological observation of *B. subtilis*. FM-4-64, di-4-ANEPPS, DilC12, FM5-95, and nonyl acridine orange have been previously utilized (Cowan et al., 2004; Kawai et al., 2004; Wen et al., 2022). Additionally, acridine orange, auramine O, 3,3′-Dihexyloxacarbocyanine Iodide (DiOC6(3)), Amino Naphthyl Ethenyl Pyridinium dye (di-4-ANEPPS), and thioflavin T have been used to analyze spore structures (Cowan et al., 2004; Magge et al., 2009; Kuwana et al., 2023). In this study, we demonstrated that safranin O staining can be a convenient tool to visualize structural differences in bacterial spores, despite it being commonly used to analyze plant tissues based on its lignin content-dependent fluorescence shifts. Our findings suggest that in bacterial cells, which lack lignin, the fluorescence properties of safranin O may be influenced by other factors. Using multiple fluorescent reagents, including APBT, auramine O, thioflavin T, and congo red, we identified distinct staining patterns, revealing previously uncharacterized spore structures. Additionally, comparative analysis with GFP-fusion proteins provided further understanding of the spatial organization of crucial spore components. The study highlights the utility of fluorescence microscopy for studying bacterial spore structures. Applying the staining procedures using the fluorescent reagents described in this study may help reveal previously unidentified spore structures.

## Data Availability

The original contributions presented in the study are included in the article/Supplementary material, further inquiries can be directed to the corresponding author.

## Supplementary material

Supplementary Figure S1 Autofluorescence of *Bacillus subtilis* 168 spores observed under fluorescence microscopy using different filter sets and exposure times. Unstained spores of B. subtilis 168 were imaged to assess intrinsic autofluorescence under conditions matching those used in this study. **(A–C)** Images obtained using **(A)** Blue filter set (U-MNUA2), **(B)** Green filter set (U-MGFPHQ), and **(C)** Red filter set (U-MWG2). For each filter set, fluorescence images were captured at increasing exposure times (from left to right: 0.25, 1, 4, 8, and 16 s). **(D)** Corresponding phase-contrast microscopy image of the same spores. Scale bar: 5 μm.

Supplementary Figure S2 Light microscopy images of *Bacillus subtilis* cells stained with safranin O under three conditions. **(A–C)** Images of cells stained with **(A)** 2.5 mg/mL (standard Gram staining concentration), **(B)** 1.0 mg/mL (100-fold concentration used in this study for fluorescence microscopy), and **(C)** 0.01 mg/mL safranin O. In condition **(A)**, cells were heat-fixed before staining. In **(B)** and **(C)**, cells were observed in suspension without fixation. Arrows indicate vegetative cells (VCs), mother cells (MCs), forespores (FSs), and mature spores (MSs). Scale bar: 5 μm.
