# Supplementary figures and images for: Combination of fluorescent reagents with 2-(4-aminophenyl) benzothiazole and safranin O was useful for analysis of spore structure, indicating the diversity of Bacillales species spores
Source: Front Microbiol. 2025 Jun 25;16:1603957. doi: 10.3389/fmicb.2025.1603957 (PMC12239749; doi:10.3389/fmicb.2025.1603957)

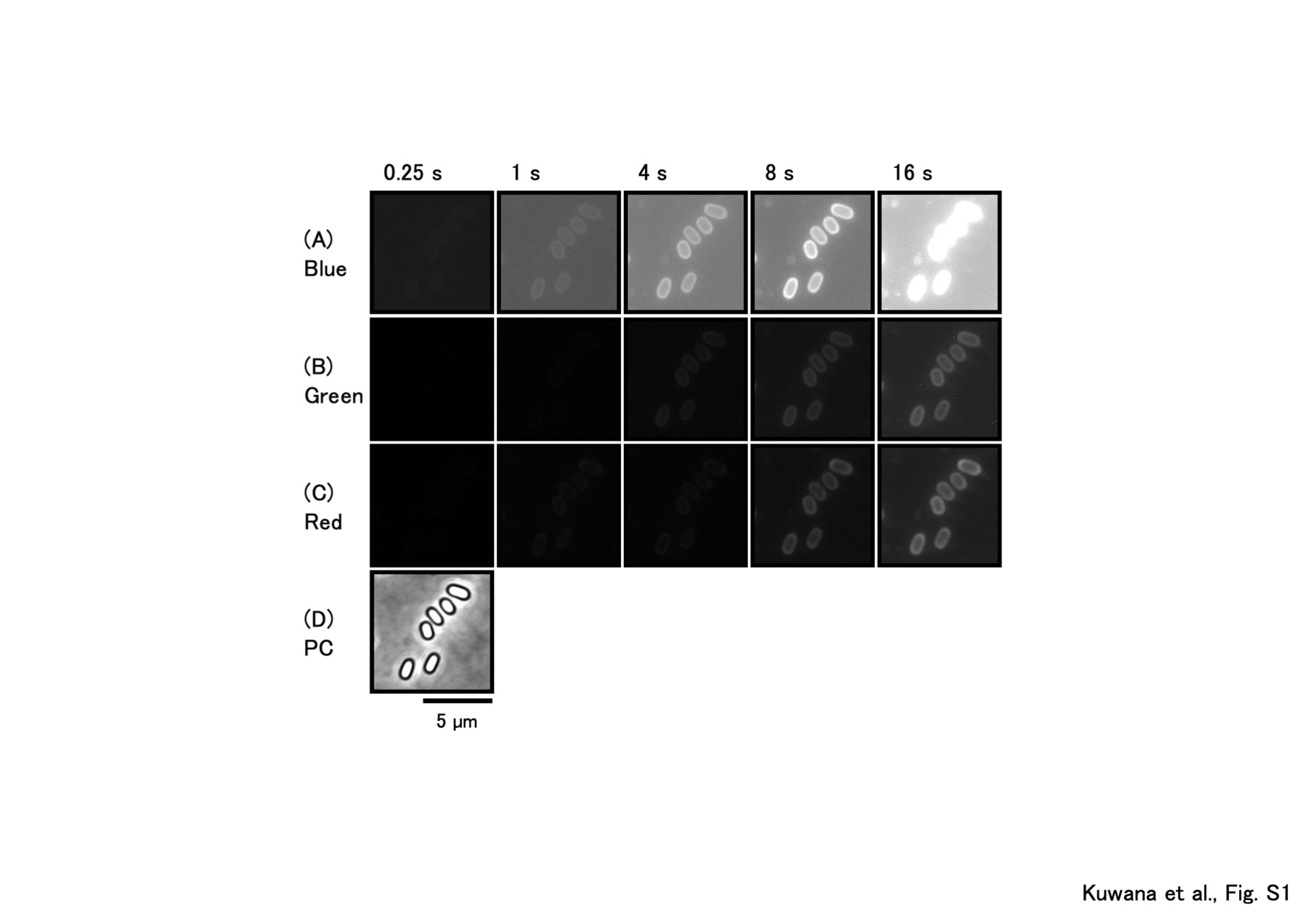

Supplement: Supplementary Figure S1 — Autofluorescence of Bacillus subtilis 168 spores observed under fluorescence microscopy using different filter sets and exposure times. Unstained spores of B. subtilis 168 were imaged to assess intrinsic autofluorescence under conditions matching those used in this study. (A–C) Images obtained using (A) Blue filter set (U-MNUA2), (B) Green filter set (U-MGFPHQ), and (C) Red filter set (U-MWG2). For each filter set, fluorescence images were captured at increasing exposure times (from left to right: 0.25, 1, 4, 8, and 16 s). (D) Corresponding phase-contrast microscopy image of the same spores. Scale bar: 5 μm. [file Image_1.jpeg]

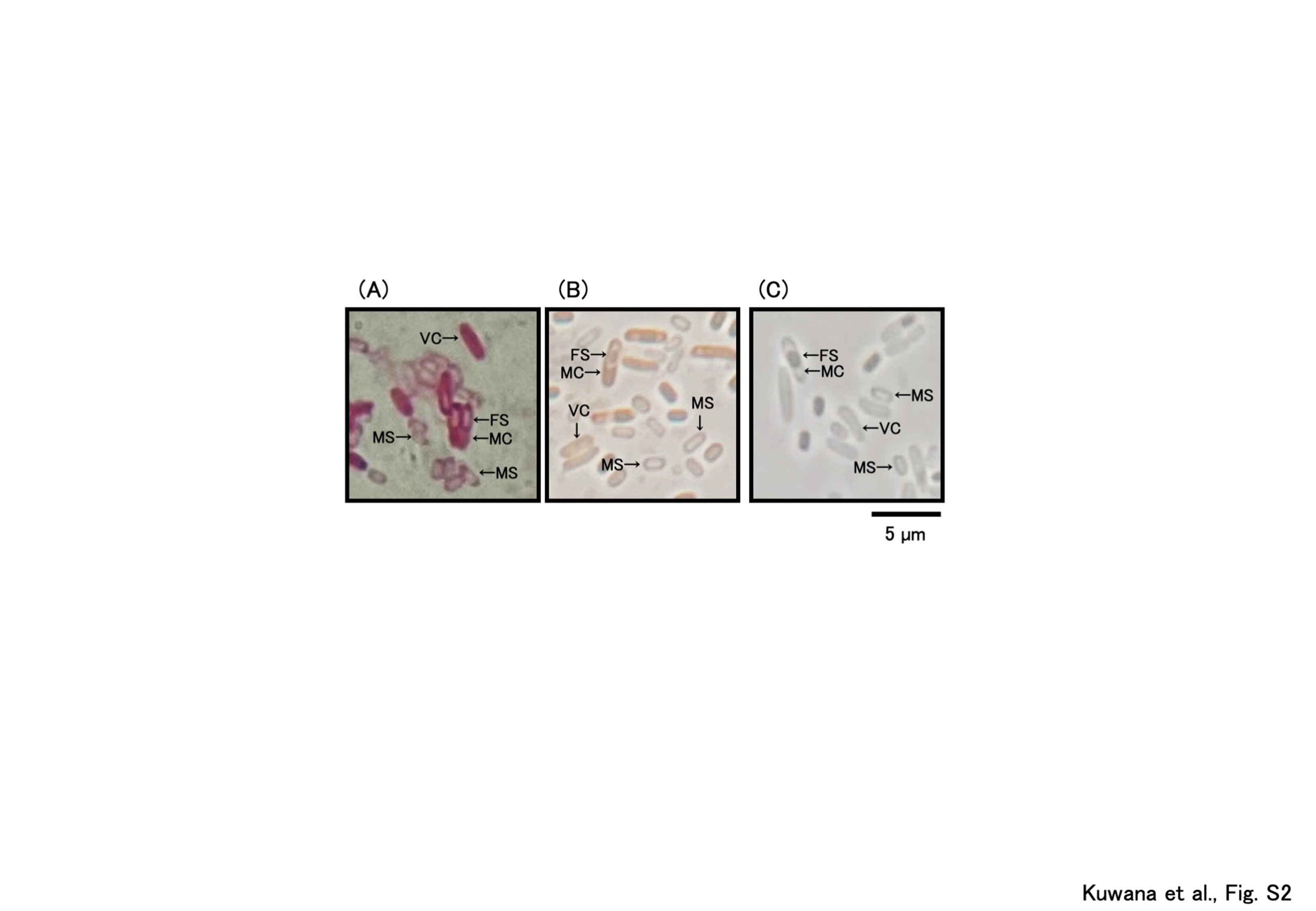

Supplement: Supplementary Figure S2 — Light microscopy images of Bacillus subtilis cells stained with safranin O under three conditions. (A–C) Images of cells stained with (A) 2.5 mg/mL (standard Gram staining concentration), (B) 1.0 mg/mL (100-fold concentration used in this study for fluorescence microscopy), and (C) 0.01 mg/mL safranin O. In condition (A), cells were heat-fixed before staining. In (B) and (C), cells were observed in suspension without fixation. Arrows indicate vegetative cells (VCs), mother cells (MCs), forespores (FSs), and mature spores (MSs). Scale bar: 5 μm. [file Image_2.jpeg]
